# First study on microscopic and molecular detection of *Acanthocheilonema reconditum* and *Leishmania infantum* coinfection in dogs in Southwest Colombia

**DOI:** 10.14202/vetworld.2023.94-103

**Published:** 2023-01-12

**Authors:** Ruben Dario Pérez-Ramírez, Rodrigo Lugo-Vargas, Julieth Michel Petano-Duque, Juan Sebastian Cruz-Méndez, Iang Schroniltgen Rondón-Barragán

**Affiliations:** 1 Research Group in Immunobiology and Pathogenesis, Laboratory of Immunology and Molecular Biology, Faculty of Veterinary Medicine and Zootechnics, Universidad del Tolima, Santa Helena Highs, Ibagué-Tolima, Colombia; 2 Poultry Research Group, Laboratory of Immunology and Molecular Biology, Faculty of Veterinary Medicine and Zootechnics, Universidad del Tolima, Santa Helena Highs, Ibagué-Tolima, Colombia

**Keywords:** *5.8s-ITS2-28s*, *Acanthocheilonema reconditum*, *COX1*, *hsp70*, *Leishmania infantum*

## Abstract

**Background and Aim:**

Canine vector-borne diseases represent an important issue for the welfare and health of animals, but also have great zoonotic potential. These diseases are caused by bacteria, nematodes such as filariae, and other parasites such as *Leishmania* spp. Given the difficulty in differentiating common microfilariae in dogs by microscopy and serological methods, molecular techniques such as polymerase chain reaction (PCR) and sequencing should be valuable for reaching a reliable diagnosis. This study aimed to use microscopy and PCR to identify the microfilarial species in dogs from Valle del Cauca, Colombia, and a possible association with *Leishmania infantum* parasites.

**Materials and Methods:**

This study was conducted on 270 dogs from Pradera and Florida municipalities. Microfilariae were detected in dogs by optical microscopy and amplification with *5.8S-ITS2-28S*. Species identification was achieved through the amplification of the gene *cytochrome oxidase I* (*COX1*).

**Results:**

Microscopic detection of microfilariae was possible in 4.81% (13/270) of the dogs. In addition, by PCR of *COX1* and Sanger sequencing of *ITS2*, *Acanthocheilonema reconditum* was identified as the circulating microfilarial species in 12 dogs, coinfecting with the species *L. infantum* (*Leishmania donovani* complex).

**Conclusion:**

To the best of our knowledge, this is the first report on *A. reconditum* and *L. infantum* mixed infection in dogs in Colombia, particularly in the Valle del Cauca.

## Introduction

Mixed infections of different parasites and bacteria in dogs have been widely reported globally, typically occurring because several pathogens are transmitted to common hematophagous arthropods [1–3]. Various studies have reported coinfection of microfilarial species with *Anaplasma* spp., *Ehrlichia* spp., and *Leishmania* spp. [2, 4–7]. These pathogens have the potential to infect humans, so canine vector-borne diseases are an important group of zoonotic illnesses for veterinary and public health [8]. Filarial worms are nematodes of the order Spirurida and superfamily Filarioidea, which infect dogs acting as definitive hosts with hematophagous arthropods acting as intermediate hosts. Dogs become infected while feeding and arthropods act as a vector for the parasite when they ingest blood [9]. The species *Dirofilaria immitis* and *Dirofilaria repens*, belong to the Dirofilariinae subfamily, are typically found in tissue spaces and body cavities, or sometimes within the blood vessels or lymphatic system. The Onchocercidae subfamily contains *Acanthocheilonema reconditum*, *Acanthocheilonema dracunculoides*, and *Cercopithifilaria* spp., which are characterized by the production of microfilariae in blood or skin [9–11]. Adult filariae of *A. reconditum* have been found in the subcutaneous tissues of the limbs, back, and perirenal fat, as well as the thoracic and abdominal cavities of dogs [9, 12]. The main vectors are fleas of the species *Ctenocephalides canis*, *Ctenocephalides felis*, *Pulex irritans*, *Pulex simulans*, and *Echidnophaga gallinacea*; as well as lice of the species *Heterodoxus spiniger* and *Linognathus setosus* [9, 13]. It has also been suggested that *C. felis* could be a mechanical vector for *Leishmania* spp., representing an alternative route for the transmission of protozoa between dogs [14].

Once *A. reconditum* colonizes subcutaneous tissues or cavities, it produces the L1 larval phase in blood from 67 to 101 days [13]. This larval phase is the focus of diagnosis by microscopy or molecular techniques [15–17]. Discrimination of *D. immitis*, *D. repens*, and *A. reconditum* is performed using microfilarial concentration techniques (e.g., Knott’s test) based on morphometric criteria, histochemical staining, and molecular techniques. Still, it can be challenging, particularly for samples from areas, where different filarioids are present in sympatry [16, 18, 19]. In Colombia, several studies have detected *D. immitis* by immunochromatographic tests and enzyme-linked immunosorbent assays [20]. However, there have been few reports on the molecular diagnosis of other filarial species, such as *A. reconditum*. On the other hand, leishmaniasis is a group of zoonotic diseases caused by protozoan parasites of the *Leishmania* genus transmitted by female sandflies [21]. Clinical manifestations of leishmaniasis in humans and other mammals include cutaneous leishmaniasis, mucocutaneous leishmaniasis, and visceral leishmaniasis [22]. These diseases have been reported in 98 countries, with an incidence of 1.3 million new cases annually, with tropical regions being particularly affected. One affected region is Colombia, in which nine *Leishmania* species have been reported [22, 23].

This study aimed to establish circulating filarial species and their possible coinfection with *Leishmania* spp. by microscopic and molecular methods in samples from dogs in the municipalities of Pradera and Florida, Valle del Cauca, Colombia.

## Materials and Methods

### Ethical approval and Informed consent

All the experimental procedures followed the Guidelines of the Bioethics Committee of the Central Office for Scientific Research and Development of the University of Tolima, based on Law 84/1989 and Resolution 8430/1993, and complied with the guidelines for animal care and use in research and teaching [24, 25]. In addition, the permission and written consent of the owners of the dogs that are part of the study population were obtained to take the samples of the dogs. Blood samples were colleccted by veterinarians who complied with regulations and guidelines on the husbandry and welfare of the animal.

### Study period and location

The study was conducted from January to June 2021 in the municipalities of Pradera and Florida, Valle del Cauca, Colombia. The samples were processed at Laboratory of Immunology and Molecular Biology of The University of Tolima.

### Sampling

Blood samples (4 mL) were taken from 270 dogs between 6:00 am and 8:00 am, and 4:00 pm and 6:00 pm. The sample size was based on the estimated population of dogs (10,300 dogs) in these municipalities, a confidence level of 95%, a margin of error of 5%, and a 50% response distribution. In Pradera municipality, 50 samples were from dogs from rural areas and Renacer Animal Foundation, and 180 were from dogs attending San Francisco de Asís Veterinary Center (urban area). Meanwhile, 40 dog blood samples were taken from the canine shelter “De Nano” located in Florida municipality. All dogs underwent clinical examination as well as clinical laboratory testing when required (e.g., hematology and blood chemistry). Blood sampling was performed by peripheral venipuncture in the saphenous and/or cephalic vein of the dog, with prior consent from the owner. Samples were placed in tubes with ethylenediaminetetraacetic acid (EDTA) anticoagulant and stored at −20°C until use.

### Clinical analysis and microscopy

A complete clinical examination was performed through physical examination to detect signs presented by the dogs at the time of sample collection. For microscopy, 52.5 μL of dog whole blood was transferred into 70 μL capillaries without heparin, after which the end of the capillary was sealed by heating and centrifuged at 13,700× *g* for 5 min (Adams Autocrit Ultra 3, BD, USA). This was followed by a complete hematic biometric examination, which included the determination of hemoglobin, hematocrit, and white and red blood cell counts, on a Genrui KT 6610 hematology analyzer (Genrui Biotech, China). Detection of filarial microorganisms and identification of morphological characteristics were performed using the Woo test (5× and 10×) [17] and the Wright staining of blood smears (100×) in an MPR 3000 binocular microscope (Scientific, USA).

### Extraction of DNA and DNA quality

Genomic DNA (gDNA) was extracted from whole-blood samples (with EDTA) of dogs previously diagnosed with microfilariae by microscopy using the EZNA^®^ Tissue DNA kit (Omega Bio-Tek, USA), in accordance with the manufacturer’s instructions. The quality of the extracted product was verified using the NanoDrop One spectrophotometer (Thermo Fisher, USA) and through the amplification of a 497 bp fragment of the canine *beta-actin* (*ACTB*) gene with primers designed in our laboratory (forward primer 5’-GGCTACAGCTTCACCACCAC-3’ and reverse primer 5’-TACTCCTGCTTGCTGATCCACA-3’) (NM_001195845.3).

### PCR detection of microfilarial species and *Leishmania* spp.

Molecular characterization of circulating microfilariae in dogs from Pradera and Florida was performed by amplifying *5.8S-ITS2-28S* ribosomal RNA (*rRNA*) of different species with differential amplicon sizes, and a fragment of the *cytochrome oxidase C* subunit 1 (*COX1*) gene from *D. immitis*, *D. repens*, *A. reconditum*, and *Cercopithifilaria* spp. (Table-1) [15, 26–30]. In addition, a fragment of the *nd5* gene of *Onchocerca* spp. was amplified in P13, as similarly occurred for a fragment of *18S rRNA* of other nematode species such as *Ascaris suum, Mansonella perstans, Mansonella ozzardi, Capillospirura ovotrichuria, Loa loa, Brugia malayi, Toxocara canis*, and *Wuchereria bancrofti* (Table-1). To identify *Wolbachia* spp., an endosymbiont bacterium, a 595 bp fragment of the *Wolbachia surface protein* (*wsp*) gene, was amplified using the primers described in Table-1.

**Table-1:** Primers sequences for amplification of microfilariae, *Wolbachia* spp., and *Leishmania* spp. genes.

Organism	Gene/Region	Primers (5’- 3’)	Ta (°C)	Amplicon size (pb)	Reference
*A. reconditum*	*COX1*	F: ATCTTTGTTTATGGTGTATC	50	589	[26]
		R: ATAAGTACGAGTATCAATATC			
*Cercopithifilaria* spp.	*COX1*	F: CGGGTCTTTGTTGTTTTTATTGC	50	304	[26]
		R: ATAAGTACGAGTATCAATATC			
*D. immitis*	*COX1*	F: ACCGGTGTTTGGGATTGTTA	50	169	[26]
		R: ATAAGTACGAGTATCAATATC			
*D. repens*	*COX1*	F: GTATAATTTTGGGTTTACATACTGTA	50	479	[26]
		R: ATAAGTACGAGTATCAATATC			
*A. reconditum* *D. immitis* *D. repens*	*5.8s-ITS2-28s* rRNA	F: AGTGCGAATTGCAGACGCATTGAG	58	577 542 484	[27]
		R: AGCGGGTAATCACGACTGAGTTGA			
*M. ozzardi* *M. perstans* *B. malayi* *T. canis* *W. bancrofti*	*18S rRNA*	F: TCGTCATTGCTGCGGTTAAA	55	1147	[15]
		R: GGTTCAAGCCACTGCGATTAA			
*Onchocerca* spp.	*nd5*	F: TTGGTTGCCTAAGGCTATGG	55	471	[28]
		R: CCCCTAGTAAACAACAAACCACA			
*Wolbachia* spp.	*wsp*	F: TGGTCCAATAAGTGATGAAGAAACTAGCTA	50	595	[29]
		R: AAAATTAAACGCTACTCCAGCTTCTGCAC			
*Leishmania* spp.	*hsp70C*	F: GGACGAGATCGAGCGCATGGT	61	234	[30]
		R: TCCTTCGACGCCTCCTGGTTG			

*Ta: Annealing temperature. *A. reconditum*=*Acanthocheilonema reconditum, D. repens*=*Dirofilaria repens,* *D. immitis*=*Dirofilaria immitis, M. ozzardi*=*Mansonella ozzardi, M. perstans Mansonella perstans, B. malayi*=*Brugia malayi, T. canis*=*Toxocara canis, W. bancrofti*=*Wuchereria bancrofti, COX1*=*Cytochrome oxidase I,* rRNA=Ribosomal RNA, *wsp*=*Wolbachia surface protein, hsp70C*=*Heat shock protein 70*

Evaluation of *Leishmania* spp. coinfection was carried out through amplification of a fragment of the C region *heat shock protein 70* (*hsp70C*) gene (Table-1) using *Leishmania infantum* (MCAN/CN/90/SC) as a positive control and gDNA from a dog negative for *Leishmania* spp. infection from a previous study at our laboratory as a negative control.

Polymerase chain reaction (PCR) was carried out in a ProFlex PCR System thermocycler (Applied Biosystems, USA), using a final reaction volume of 25 μL, composed of 14.85 μL of distilled and deionized water, 5 μL of 5× OneTaq Standard Reaction Buffer, 2 μL of 1.5 mM dNTPs, 1 μL of each primer (forward and reverse) [10 pmol/μL], 0.15 μL of OneTaq^®^ DNA Polymerase (New England BioLabs, Madison, USA), and 1 μL of total DNA sample. Amplification consisted of initial denaturation at 94°C for 3 min; 35 cycles of denaturation at 94°C for 30 s, annealing at different temperatures (Table-1) for 30 s, and extension at 68°C for 30 s for all reactions; except for the amplification of the *5.8S-ITS2-28S* rRNA fragment in which 1 min was used; and final extension at 68°C for 5 min.

Polymerase chain reaction products were revealed by horizontal electrophoresis in 2% agarose gel, staining with HydraGreen (ACTGene, USA), placement in a myGel Mini electrophoresis chamber (ACCURIS, USA) for 40 min at 100 V, and visualization under ultraviolet light using an ENDURO GDS™ gel documentation system (Labnet Intl, USA).

### DNA sequencing

Positive PCR products of the *5.8S-ITS2-28S* rRNA of microfilariae and two PCR products of *hsp70C* of *Leishmania* spp. from dog were confirmed by Sanger sequencing (Macrogen, Korea). Reads were compared with those reported in the database GenBank using the BLAST tool. Obtained sequences were assembled with Geneious Prime software version 2022.0.1 [31]. Revised sequences of microfilarial species were reported to GenBank with accession numbers MZ473246, MZ473247, MZ473248, MZ473249, MZ468150, MZ468151, and MZ474199. In the same way, detected sequences from *L*. *infantum* were reported with the accession numbers MZ605427 and MZ605428.

### Phylogenetic analysis

To represent the genetic relationship between the microfilariae detected in Pradera and Florida (Valle del Cauca), a phylogenetic tree was created based on the *5.8S-ITS2-28S* rRNA sequences obtained in this study and sequences previously reported in GenBank. The method used was neighbor-joining, with 1000 iterations in Geneious Prime software version 2022.0.1 [31]. Sequences of *A. reconditum* and representative microfilarial species were used. In addition, *Ascaridia galli* (*Ascarididae* and *Ascaridida*) was selected as an outgroup.

## Results

### Microscopy identification

**Figure-1:**
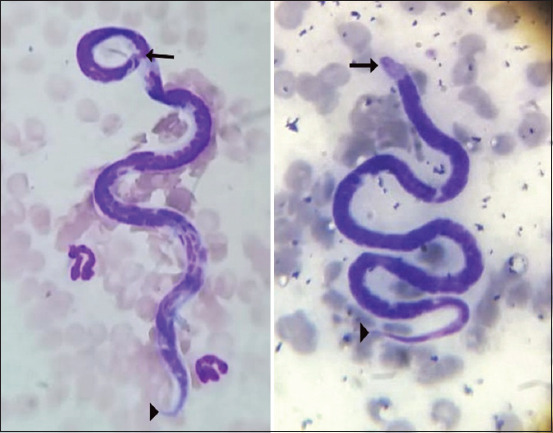
Microfilariae in peripheral blood smear. The cephalic region (arrow) is evident, as well as the sharp tail (arrowhead). Wright stain, 100×.

The Woo test and Wright staining allowed the detection of microfilariae in 4.81% (13/270) of dog blood smears. The cephalic region was evident, as well as the filiform (sharp) ending of the caudal part (Figure-1). Progressive and stationary movements were also observed. The observations corresponded to the morphological features of *D*. *immitis*, *D*. *repens*, and *A*. *reconditum*.

### Clinical and hematological observations

In our study, 53.85% (7/13) of the dogs positive for microfilariae by microscopy were male (Table-2). Meanwhile, 46.15% and 53.85% lived in rural and urban areas, respectively (Table-2). In addition, 76.92% of dogs (10/13) positive for microfilariae showed clinical signals, while 92.30% of dogs (12/13) presented fleas and ticks (Table-2). Additional clinical characteristics of the dogs are shown in Table-2.

**Table-2:** Clinical characteristics of the *Leishmania*-positive dogs from Pradera and Florida Valle del Cauca.

Patient	Locality	Gender*	Age (years)	Zone	Body condition (1–5)	Ectoparasites (fleas and ticks)	Clinical signals	Live with more animals
P1	Pradera	M	16	Rural	2	+	Lethargy, loss of appetite, joint disease	-
P2	Pradera	M	10	Rural	4	+	Conjunctivitis, eye secretion	+
P3	Pradera	F	9	Urban	3,5	-	-	+
P4	Pradera	F	6	Urban	1	+	Dehydration, cachexia, loss of appetite, pale mucous membranes, vomiting 3 times a day. Polydipsia	+
P5	Pradera	F	6	Urban	3,5	+	Onychogryphosis in the right pelvic limb, lymphadenopathy, periorbital skin dermatitis, left eye, and nasal plane. Flies	+
P6	Pradera	M	10	Urban	3,5	+	Pinna dermatitis, fore and hind limb dermatitis, lymphadenopathy	+
P7	Pradera	M	4	Urban	4	+	-	+
P8	Pradera	M	3	Urban	3	+	-	+
P9	Pradera	F	3	Rural	2,5	+	Pale mucous membranes, lymphadenopathy, ventral dermatitis, petechiae, lethargy	+
P10	Pradera	F	5	Urban	3	+	Bilateral left dry eye, dermatitis, pale mucous membranes	+
P11	Florida	F	1	Rural	3	+	-	+
P12	Florida	M	2	Rural	4	+	-	+
P13	Florida	M	5	Rural	2	+	-	+
P14	Pradera	F	12	Rural	3,5	+	-	+

Leukocytosis, thrombocytopenia, lymphocytosis, and eosinophilia were identified among the hematological alterations found in the different dogs. Anemia was found in 46.15% (6/13) of the dogs.

### Molecular detection

**Figure-2:**
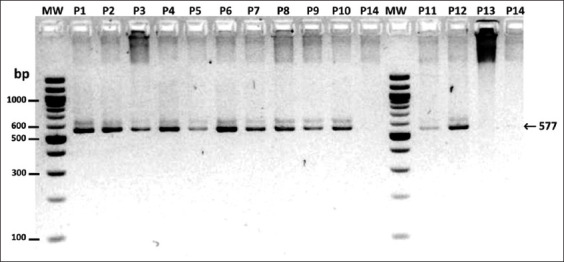
Amplification of *5.8s-ITS2-28s* rRNA from filariae in samples from dogs in Florida and Pradera municipalities of Valle del Cauca, Colombia. MW=Molecular weight marker 100 bp DNA ladder (New England Biolabs, USA), P1–P13=Microfilaremic dogs, P14=No microfilaremic dog. Agarose gel 2%.

All samples generated an amplicon of approximately 497 bp corresponding to the canine *ACTB* gene (data not shown), indicating adequate extraction and gDNA quality. By amplification of *5.8S-ITS2-28S* rRNA, it was determined that the possible microfilarial species in the 12 positive dogs were *A. reconditum* and *D. immitis*, whose amplicon sizes are similar, while one dog was negative for microfilarial species (Figure-2). The 12 samples positive for microfilariae were positive for *A. reconditum* and negative for *D. immitis*, *D. repens*, and *Cercopithifilaria* spp., as determined through amplification of the *COX1* gene using species-specific primers.

Under our conditions, amplicons for *Wolbachia* spp. were not detected in any dog. In addition, in the sample negative for microfilarial species, no other possible nematodes (e.g., *Onchocerca* spp. and *Mansonella* spp.) were found through PCR using the primers described in Table-1.

**Figure-3:**
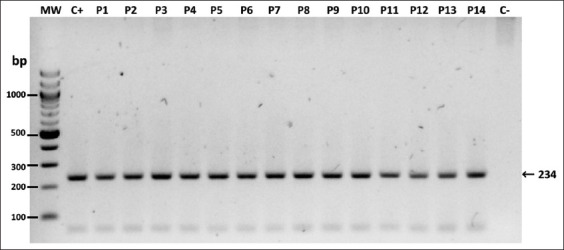
Phylogenetic tree of sequences of microfilaria species using *ITS2*. A bootstrapped NJ tree was constructed using Geneious Prime software (Version 2021.1.1). Bootstrap consensus tree inferred from 1000 iterations, where the numbers in the branches correspond to the percentage of times the node appears in the 1000 iterations. Sequences (Accession numbers) used for the tree are as follows: *Acanthocheilonema reconditum* (P1, MZ473246), *A. reconditum* (P2, MZ473247), *A. reconditum* (P4, MZ473248), *A. reconditum* (P6, MZ473249), *A. reconditum* (P10, MZ468150), *A. reconditum* (P11, MZ474199), *A. reconditum* (P12, MZ468151), *A. reconditum* (KX932127), *Acanthocheilonema dracunculoides* (KP420152), *Dirofilaria repens* (JX524743), *Dirofilaria immitis* (MG189966.1), *Cercopithifilaria* spp. (JF501396.1), and *Ascaridia galli* (LC592812).

Meanwhile, all positive dogs were positive for the amplification of a fragment of the C region of the *hsp70* gene from *Leishmania* spp. (Figure-3). Sequencing of PCR products for *Leishmania* spp.-positive dogs from Pradera and Florida (MZ605427 and MZ605428) showed a match with *L. infantum* with 99.57% identity with *Leishmania* *donovani* complex.

### ITS2 sequencing and phylogenetic analysis

**Figure-4:**
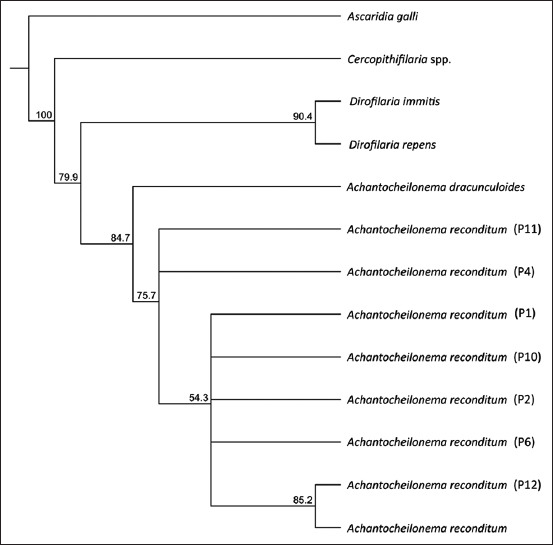
Amplification of *heat shock protein 70* gene fragment (234 bp) of *Leishmania* spp. in samples from microfilaremic dogs in Florida and Pradera municipalities of Valle del Cauca, Colombia. MW=Molecular weight marker, 100 bp DNA ladder (New England Biolabs, USA), C+=Positive control, *L. infantum* (MCAN/CN/90/SC), C-=Negative control, P1–P13=Microfilaremic dogs, P14=No microfilaremic dog. Agarose gel 2%.

All the samples were identified as *A. reconditum* with identity values ranging from 83.1% to 99.8%. Phylogenetic analysis of the microfilariae showed well-defined and separated branches that agree with different representative microfilarial species and *A. dracunculoides* as the sister group of *A. reconditum* clade (Figure-4). The sequences of *A. reconditum* in this study formed two polytomies in the same clade, in which there is also a branch made up of a sequence from this study and a sequence reported in GenBank (Figure-4).

## Discussion

Filariasis is a parasitic disease found globally in both wild and domestic animals, as well as humans. For its epidemiological surveillance, it is necessary to differentiate potential etiologic agents [32]. Laboratory diagnosis of infection caused by *D. immitis*, *D. repens*, or *A. reconditum* is achieved through classical detection of circulating microfilariae, parasite antigens, and/or nucleotides [16, 26].

To identify microfilariae in the bloodstream of infected animals, microscopic techniques as Woo’s test and Knott’s test can be used [9, 16, 33, 34]. Knott’s test is the gold standard [19], while key diagnostic features include differences in morphology and size of particular structures. However, there may be a lack of sensitivity in microscopic detection of circulating microfilariae. Direct smear and Woo’s test have been reported not to be sensitive when there is a low number of microfilariae (50 mfs/mL), although Courtney and Zeng [35] detected them at a level of <10 mfs/mL. Despite this, the sensitivity of a direct smear may be 20% lower than that presented by Knott’s test, namely, 1 mfs/mL [35]. In addition, blood-circulating microfilariae of *D. immitis* should be discriminated from those of other filarioids that do not infest the heart chambers and arteries (i.e., *D. repens* and *A. reconditum*).

The differential diagnosis of circulating microfilariae is based on morphological characteristics such as the shape of the cephalic hook and the posterior part and length and diameter of the parasite body. However, discrepancies have been reported in the measurements obtained for identifying filarial organisms using this technique [9, 11, 16], which may be due to the techniques used to perform staining and/or fixation [36].

In this study, microscopic observations were similar to the features of *D. immitis*, *D. repens*, and *A. reconditum* reported by McCall *et al*. [19] and Otranto *et al*. [11] (Figure-1). The similar morphology of these species makes the differentiation difficult, leading to misdiagnosis [37, 38]. Besides, both *A. reconditum* and *D. immitis* exhibit the same characteristic movements [39]. Thus, to discriminate microfilarial species, there is a need for additional techniques such as histochemistry (e.g., acid phosphatase staining) or molecular methods (endpoint PCR and PCR-Ribotyping) [16]. Furthermore, since the treatment of *A. reconditum* infection differs from that of heartworm infection, it is important to differentiate between the two parasites [37].

The sex distributions of dogs positive for microfilariae as determined by microscopy were reported by Cringoli *et al*. [40] and Theis *et al*. [41], who described that males are more susceptible to *A. reconditum* infections than females. It has also been reported that *A. reconditum* is present at a higher prevalence in older dogs [40, 41]; however, here, dogs with microfilaremia ranged in age from 1 to 16 years old (Table-2).

In contrast to other filarioids transmitted by mosquitoes (e.g., *D. immiti*s and *D. repen*s) to dogs, fleas, and lice are vectors of *A. reconditu*m in dogs, where the rate of infestation by fleas is about 5% [12, 32]. This is in line with the results obtained in this study, since the dogs evaluated here presented fleas. Nonetheless, ticks were also found, which are a vector that transmits *A. dracunculoides* and *Cercopithifilaria* spp. [32].

At the time of sampling, the dogs positive for microfilariae were clinically healthy. In this regard, Engelmann *et al*. [37] reported the presence of *A. reconditum* without specific clinical signs and mentioned that parasites were detected incidentally during routine laboratory examinations. In addition, during intra-abdominal surgical procedures or among subcutaneous tissues, adult worms have been found to live in the peritoneal cavity, causing nodule formation [32]. Notably, *A. reconditum* is a species for which treatment is not usually required because its identification allows the avoidance of inappropriate medical treatment [27]. Despite animals with *Acanthocheilonema* spp. being of minimal veterinary clinical significance, all dogs with filariae have the potential to infect humans and remain important to public health [11, 42]. The treatment can be indicated to reduce the risk of transmission and avoid the appearance of new cases [11].

It should be highlighted that, in our study, 3.85% (7/13) of dogs showed lymphadenopathy, loss of appetite, conjunctivitis, eye secretion, and dermatitis in different anatomical sites, such as periorbital skin, ear, and ventral abdomen wall (Table-2). Regarding this, dermatitis has been suggested as a clinical sign of *A. reconditum* infection [12]. In the same way, the clinical signs most frequently found in dogs positive for *Leishmania* spp. include dermatological signs, lymphadenomegaly, weight loss, and ocular signs [43].

Leukocytosis with eosinophilia has been reported in dogs with microfilaremia by *A. reconditum* [44, 45], which agrees with the hematological alterations in the two dogs. According to Hashem and Badawy [44], lymphocytosis, which was found in P4, P8, and P10, could be related to a chronic phase of filariasis accompanied by normochromic normocytic anemia. In addition, the increase in plasma proteins with leukocytosis, eosinophilia, and lymphocytosis may be related to the immune response of the dog to circulating microfilarial antigens [44, 45]. Thrombocytopenia observed in P2, P6, P7, P8, and P10 may be associated with other vector-borne diseases (*Ehrlichia canis*, *Anaplasma phagocytophilum*, or *Anaplasma platys*, and *Leishmania* spp.), for which mild-to-moderate symptoms are very common [46].

In addition, in this study, anemia was found in microfilaremia-positive dogs, but those testing negative also had mild anemia, suggesting other potential causes of the anemia, including vector-borne diseases not considered in this study. In accordance with this, many symptoms attributed to this parasite are commonly the outcome of concurrent parasitism [5]. However, mild-to-moderate normocytic normochromic anemia is the most common hematologic change in canine leishmaniasis [46].

In our study, two dogs had high serum creatinine levels (8 and 2.99 mg/dL). Although kidney is not a target organ for microfilarial infection, some reports have shown proteinuria and hematuria as common findings [47, 48]. In the case of creatinine, it has been reported that creatinine levels increase due to microfilarial infection in humans and canines [49, 50], which may be caused by kidney dysfunction and intravascular hemolysis associated with infection as well as immune-mediated glomerular damage, mainly in chronic infection [51, 52]. In addition, the deposition of circulating immune complexes in the glomerulus produced by various diseases, including leishmaniasis, leads to chronic nephropathy, characterized by glomerulosclerosis, renal hypertension, and tubulointerstitial nephritis [46].

In comparison with serological methods and microscopy, PCR and molecular analyses are highly sensitive for the detection of microfilariae, simultaneous diagnosis, identification of the genetic relationships between and within species, and providing more reliable data for clinical and epidemiological purposes [18, 26, 27, 38, 53]. In molecular diagnosis, the *COX1* gene has been used, allowing filarial species diversity to be determined [54]; meanwhile, *5.8S-ITS2-28S* rRNA has been used for the identification of microfilarial species commonly found in dogs, eliminating the need for multiple assays, and different methods for recognizing species observed in blood smears [27]. In addition, ITS2 has better sensitivity, specificity, and capacity for species identification than ITS1 in samples with low microfilaremia (<2.5 mfs/10 μL) [38].

Regarding the lack of amplification of IST2 in a dog positive for microfilariae, a previous study found that the absence of expected bands is associated with the detection limit of PCR [26]. In this regard, Latrofa *et al*. [26] reported a detection limit for *A. reconditum* by PCR of 8 mfs/mL. It should be noted here that, in this study, the level of microfilariae per milliliter was not measured, but in future studies, microfilariae of each sample should be quantified. In addition, *A. reconditum* has a global distribution and, in many geographical areas of the Mediterranean Basin, Middle East, South Africa, South America, and Oceania, it is the sole or the most prevalent filarioid species infesting dogs [12].

However, our results of ITS2 sequencing and phylogenetic analysis suggested that the ITS2 region is highly conserved within this species and allows differentiation from other microfilarial species (Figure-4). In addition, the distribution of the taxa in the tree has congruence with the taxonomic classification of representative microfilariae and *A. reconditum*. The findings mentioned above are in accordance with the previous studies showing homology of ITS2 sequences from *A. reconditum* parasites from the same geographical region and indicate that this region can be used as a reliable marker for the phylogenetic relationships of microfilariae [55, 56].

Notably, several studies have reported coinfection with other filariae such as *D. immitis* and *D. repens* or *D. repens* and *A. reconditum* [7, 15, 18, 57–59]. However, amplification of *COX1* ruled out the possibility of infection or coinfection with *D. immitis*, *D. repens*, or *Cercopithifilaria* spp. Meanwhile, studies have revealed polyparasitism coinfection of *W. bancrofti* and *L. donovani* [4], *W. bancrofti* and *M. perstans* with malaria and *L*. *major* [60, 61], as well as *D. immitis*, *D. repens*, *A. dracunculoides*, and *L. infantum* [6, 7]. This agrees with our results since all dogs found to be positive by microscopy were positive for *L. infantum* through the amplification of a highly conserved C region of the *hsp70* gene with six or seven *hsp70-I* copies and one *hsp70-II* copy [62, 63] (Figure-4). To the best of our knowledge, this is the first report of *A. reconditum* and *L. infantum* coinfection. Awareness of canine coinfections is an important clinical and diagnostic issue as they might induce more severe clinical signs compared with each of the microorganisms alone [7, 64].

Several studies have reported the presence of *Wolbachia* spp. in the body of filarial microorganisms (*Onchocerca volvulus*, *Onchocerca ochengi*, *D. immitis*, *D. repens*, *Litomosoides sigmodontis*, *B. malayi*, *Brugia pahangi*, *W. bancrofti*, and *M. ozzardi*) [65–68]. However, amplification of the *wsp* gene was negative in all samples, ruling out the presence of endosymbiont bacteria *Wolbachia* spp. This agrees with the findings of Ionică *et al*. [18], who did not find a correlation between *A. reconditum* and *Wolbachia* spp.

## Conclusion

To the best of our knowledge, this is the first report describing a mixed infection with *A. reconditum* and *L. infantum* in dogs in Colombia, particularly in the Valle del Cauca. The use of molecular biology methods such as PCR, contributes to our understanding of the presence of canine pathogens and extends information about the distribution of *A. reconditum* and *L. infantum* in two Colombian municipalities. The obtained results highlight the need to reinforce active public health surveillance programs, including that of dogs as a reservoir of zoonotic pathogens.

## Authors’ Contributions

RDP, RL, JP, JSC, and ISR: Conceptualization. RDP, RL, JP, JSC, and ISR: Methodology. MP, JSC, and ISR: Validation. RDP, RL, JMP, JSC, and ISR: Formal analysis. ISR: Resources. JMP and JSC: Data curation. RDP, RL, JMP, and JSC: Writing – original draft preparation. JMP, JSC, and ISR: Writing – review and editing. ISR: Supervision. ISR: Project administration. ISR: Funding acquisition. All authors have read and approved the final manuscript.

## Data Availability

The supplementary data can be available from the corresponding author on a reasonable request.
